# How does living with HIV impact on women's mental health? Voices from a global survey

**DOI:** 10.7448/IAS.18.6.20289

**Published:** 2015-12-01

**Authors:** Luisa Orza, Susan Bewley, Carmen H Logie, Elizabeth Tyler Crone, Svetlana Moroz, Sophie Strachan, Marijo Vazquez, Alice Welbourn

**Affiliations:** 1 ATHENA Network, London, UK; 2 Salamander Trust, London, UK; 3 Women's Health Academic Centre, King's College London, London, UK; 4 Factor-Inwentash Faculty of Social Work, University of Toronto, Toronto, ON, Canada; 5 ATHENA Network, Seattle, WA, USA; 6 Eurasian Women's Network on AIDS, Kiev, Ukraine; 7 UNAIDS Dialogue Platform, London, UK

**Keywords:** HIV, women, human rights, gender-based violence, mental health, values and preferences, survey, intimate partner violence, evidence base

## Abstract

**Introduction:**

Women living with HIV experience a disproportionate burden of mental health issues. To date, global guidelines contain insufficient guidance on mental health support, particularly regarding perinatal care. The aim of this article is to describe the extent and impact of mental health issues as experienced by women living with HIV on their sexual and reproductive health and human rights (SRH&HR).

**Methods:**

A global, mixed-methods, user-led and designed survey on SRH&HR of women living with HIV was conducted using snowball sampling, containing an optional section exploring mental health issues. Statistical quantitative data analysis included descriptive statistics, correlation and multiple linear regression analysis for the mental health responses. Thematic analysis of open free-text responses was performed for qualitative data.

**Results:**

A total of 832 respondents from 94 countries participated in the online survey with 489 responses to the optional mental health section. Of the respondents, 82% reported depression symptoms and 78% rejection. One-fifth reported mental health issues before HIV diagnosis. Respondents reported experiencing a 3.5-fold higher number of mental health issues after diagnosis (8.71 vs 2.48, *t*[488]=23.00, *p*<0.001). Nearly half (*n*=224; 45.8%) had multiple socially disadvantaged identities (SDIs). The number of SDIs was positively correlated with experiencing mental health issues (*p*<0.05). Women described how mental health issues affected their ability to enjoy their right to sexual and reproductive health and to access services. These included depression, rejection and social exclusion, sleep problems, intersectional stigma, challenges with sexual and intimate relationships, substance use and sexual risk, reproductive health barriers and human rights (HR) violations. Respondents recommended that policymakers and clinicians provide psychological support and counselling, funding for peer support and interventions to challenge gender-based violence and to promote HR.

**Conclusions:**

Interventions addressing intersecting stigmas and any especial impacts of diagnosis during pregnancy are required to ensure women's SRH&HR. Global policy guidelines regarding women living with HIV must incorporate mental health considerations.

## Introduction

Worldwide, about 50 to 55% [1,2] of adults with HIV are women. Mental health is core to wellbeing [3–5]. Yet, despite PubMed citing nearly 800 peer-reviewed articles on mental health in relation to women with HIV [6], this issue is either lacking [7] or has been insufficiently addressed [8–12] in global policy guidelines.

As for all women, those with HIV experience reciprocal effects of mental health on gender-based violence (GBV) [13], their sexual and reproductive health and human rights (SRH&HR) [8] and their capacity to access and adhere to healthcare and medication, when required [14,15]. These intersecting issues are not addressed comprehensively in any one guideline. Given that psychiatric symptoms, particularly depression, and mental health vulnerabilities (e.g. maladaptive coping skills) are widespread among pregnant women living with HIV [16], it is especially concerning that mental health does not feature in global perinatal transmission guidelines [7]. Even women living with HIV, who have long recognized that HIV affects them in many different, reciprocal and gendered ways [17], have rarely addressed mental health.

The World Health Organization's (WHO) Department of Reproductive Health and Research commissioned a user-led and -designed “values and preferences” global consultation to enable policymakers to address the SRH&HR of women living with HIV as part of their guideline update process [18]. The aim of this article is to describe the extent and impact of mental health issues as experienced by women living with HIV on their SRH&HR.

## Methods

### Community consultation

A pre-survey exercise led by a global reference group (GRG) identified mental health as a key issue. A survey was developed and pilot-tested by the 14 GRG members in English with iterative feedback.

### Survey

The research involved implementation of a WHO global values and preferences survey that aimed to understand access to services and priority issues among women living with HIV. The survey was distributed in seven languages and translated back into English where necessary. All sections included open-ended responses [19]. One optional section (of eight; Table 1) explored mental health issues using simply understood common terms rather than formal instruments and inquiring when respondents had experienced these issues in relation to their HIV diagnosis [19]. Answer options were not exclusive. Women were also asked to describe in free text how these mental health disorders affected their SRH&HR and how they felt that women living with HIV could best be supported to accommodate or overcome them.

**Table 1:** Content and language dissemination of the online values and preferences survey to update the WHO SRH&HR guidelines

A. Mandatory section, including experience of accessing services, priority issues	

B. Eight optional section themes	
1	Healthy sex life
2	Pregnancy and fertility
3	Violence against women living with HIV
4	Mental health issues
5	Women living with HIV in all of our diversity
6	Puberty, menstrual issues and menopause
7	HIV treatment and side effects
8	Financial issues affecting access to services

The online survey was disseminated in English, French, Spanish, Russian, Portuguese, Bahasa Indonesian and Chinese. SRH&HR, sexual and reproductive health and human rights.

### Participants

The survey study population included any woman around the world living with HIV. The process ran from February to June 2014, building on a non-random snowball sampling model developed by the ATHENA Network [20], advertised and promoted through regional and global listservs of networks of women living with HIV and clinical networks.

### Consent

The anonymous, confidential online survey began with an explanation of the questionnaire aims and purposes, including an overview of the structure and question areas, and definitions of SRH&HR [19]. Respondents were shown the following message: “I understand that by filling in the survey, I give my consent for my responses to be used in these publications. Please click agree to be able to continue.” Respondents who did not click “I agree” were unable to proceed, and their responses were not counted. Participation was taken as implicit consent. For in-person focus groups, respondents gave explicit written or verbal consent before the discussion began. Respondents were informed that they could opt to discontinue at any time or choose not to answer any individual question.

### Ethical considerations

Institutional review board approval for this survey was not sought, after discussion with the WHO Reproductive Health and Research Department and members of the Guidelines Review Committee, as this was a consultative element of the guidelines development process. Ethical considerations were undertaken in line with the WHO 2001 *Ethical and Safety Recommendations for Research on Domestic Violence against Women* and the International Community of Women Living with HIV/AIDS 2004 guidelines on involving women living with HIV in research [21,22].

### Data collection and analyses

The qualitative and quantitative data analyzed were from the online survey. Descriptive analyses were conducted to determine frequencies, mean values and standard deviations for variables. When answers were not exclusive, frequencies were derived based on the number of respondents who had never experienced the issues or who answered “don't know.” Bivariate correlations, *t*-tests and chi-square analyses were conducted to assess associations between mental health issues, socio-demographic variables and social identities. To examine the relationship between socially disadvantaged identities (SDIs) and mental health issues of respondents, a variable named *social identity index* was created, by summing identity categories to which the respondents referred themselves (Table 2). Two identities (heterosexual; married or in a stable relationship) were not considered socially disadvantaged and thus were excluded from analyses of SDI. Multiple regression analyses were performed to investigate the associations among socio-demographic variables, SDIs and mental health issues experienced before and after (since or because of) HIV diagnosis.

**Table 2:** Respondents’ demographic information

Variables	Valid sample (*n*=766)	With an answer(s) in the mental health section (*n*=489)	Without an answer(s) in the mental health section (*n*=278)	Difference tests
Age				
15 to 24	162 (21.4%)	95 (19.4%)	67 (24.1%)	
25 to 34	288 (37.6%)	173 (35.4%)	115 (41.4%)	
35 to 44	211 (27.5%)	150 (30.7%)	62 (22.3%)	
45 to 54	97 (12.7%)	66 (13.5%)	31 (11.2%)	
55 to 64	8 (1%)	5 (1%)	3 (1.1%)	
Mean age (SD)	32.98 (9.61)	33.64 (0.43)	31.81 (0.58)	*t*=2.55*
Language of respondents				
English	385 (50.3%)	258 (52.8%)	127 (45.7%)	*χ* ^2^=20.92**
Indonesian	19 (2.5%)	11 (2.2%)	8 (2.9%)	NS
Chinese	44 (5.7%)	16 (3.3%)	28 (10.1%)	NS
Portuguese	20 (2.6%)	11 (2.2%)	10 (3.6%)	NS
French	36 (4.7%)	22 (4.5%)	14 (5.0%)	NS
Spanish	86 (11.2%)	63 (12.9%)	23 (8.3%)	NS
Russian	176 (23.0%)	108 (22.1%)	68 (24.5)	NS
Social identity index				
1. I do or have done sex work.	101 (13.2%)	66 (13.5%)	35 (12.6%)	NS
2. I inject/use or have injected/used drugs.	136 (17.7%)	102 (20.9%)	34 (12.2)	*χ* ^2^=8.92**
3. My sexual partner(s) injects/uses or has injected/used drugs.	164 (21.4%)	107 (21.9%)	57 (20.5%)	NS
4. I am a client of opioid substitution therapy programme.	26 (3.4%)	20 (4.1%)	6 (2.2%)	NS
5. I am/have been in prison.	50 (6.5%)	35 (7.2%)	15 (5.4%)	NS
6. I am/have been in a detention centre.	52 (6.8%)	32 (6.5%)	20 (7.2%)	NS
7. I am living with one or more disabilities.	101 (13.2%)	77 (15.7%)	24 (8.6%)	*χ* ^2^=7.75**
8. I have or have had active TB.	99 (12.9%)	69 (14.1%)	30 (10.8%)	NS
9. I have or have had hepatitis C.	157 (20.5%)	107 (21.9%)	50 (18.0%)	NS
10. I have or have had malaria.	119 (15.5%)	96 (19.6%)	23 (8.3%)	*χ* ^2^=17.30***
11. I migrated from one country to another for economic reasons.	59 (7.7%)	45 (9.2%)	14 (5.0%)	*χ* ^2^=4.28*
12. I migrated from one country to another for political reasons.	17 (2.2%)	8 (1.6%)	9 (3.2%)	NS
13. I am lesbian, bisexual or have sex with women.	37 (4.8%)	20 (4.1%)	17 (6.1%)	NS
14. I am a trans woman.	29 (3.8%)	17 (3.5%)	12 (4.3%)	NS
15. I am a heterosexual woman.	462 (60.2%)	299 (61.1%)	163 (58.6%)	NS
16. I am married or in a stable relationship.	361 (47.1%)	229 (46.8%)	133 (47.8%)	NS
17. I am intersex.	4 (0.5%)	1 (0.2%)	3 (1.1%)	NS
18. I have experienced any form of female genital cutting or mutilation, including circumcision.	39 (5.1%)	28 (5.7%)	11 (4.0%)	NS
19. I am or have been homeless.	78 (10.2%)	55 (11.2%)	23 (8.3%)	NS
20. I am an indigenous woman.	62 (8.1%)	51 (11.4%)	11 (4.0%)	*χ* ^2^=9.92**

NS, not significant; TB, tuberculosis;

* *p*<0.05,

** *p*<0.01,

*** *p*<0.001;

*χ* ^2^, chi-squared; *t*, student *t*-test.

A mental health outcome index was created by summing the mental health issues that respondents reported experiencing. The options “since” and “because of my diagnosis” were combined into one variable to indicate the occurrence of a mental health issue following HIV diagnosis. Respondents who reported experiences both before *and* after acquiring HIV were categorized as experiencing “persistent” mental health issues.

Thematic analysis of open-ended survey responses was performed by two social science investigators (LO, CL) [23]. Transcripts were read several times and investigators made note of initial thoughts and ideas in the data; first-level codes were then developed by highlighting relevant quotes about key areas of interest (e.g. depression). Codes were collated across data sets to produce themes that highlighted similar experiences among respondents (e.g. stigma and mental health). Themes were then structured into a thematic map (e.g. the convergence of stigma and mental health issues to compromise sexual and reproductive health (SRH)) and refined (e.g. recommendations created for supporting the mental health of women living with HIV across the micro-, meso- and macro-levels).

## Results

A total of 1173 respondents were recorded on the online survey system, but 341 did not meet the criteria of giving consent and confirming HIV status. Thus there were 832 online respondents from 94 countries.

### Quantitative findings

As 66 of 832 respondents did not give their age, the final sample for quantitative analysis was 766. A further 278 (36.3%) respondents did not answer any questions in the optional mental health section so were not entered into subsequent analyses, resulting in a sample of *n*=489 for correlation and regression analyses. The early drop-outs were found to be younger (*t*=2.55, *p*<0.05) and less likely to speak English (*χ* ^2^=20.92, *p*<0.01), use drugs (*χ* ^2^=8.92, *p*<0.01), have a disability (*χ* ^2^=7.75, *p*<0.01), have had malaria (*χ* ^2^=17.30, *p*<0.001) or be economic immigrants (*χ* ^2^=4.28, *p*<0.05). Respondents’ information on age, language and social identities are presented in Table 2.

Respondents’ experiences of mental health issues are shown in Table 3. Approximately one-fifth had experienced depression (23.9%), low self-esteem (22.5%), body image issues (18.5%), feelings of rejection (18.1%) and substance use (22%) before receiving their HIV diagnosis. Paranoia (5.0%) and anorexia (5.5%) were relatively uncommon. HIV diagnosis was identified as a critical point for mental health issues, where around 70% of respondents reported experiences of depression (74.0%), shame (70.8%), self-blame (70.0%), feelings of rejection (69.9%) and insomnia (68.4%), among others. Compared to the rates before HIV diagnosis, problems occurring “since/because of” HIV diagnosis showed considerable increases across all mental health issues, ranging from 1.2-fold (substance use) to 6.7-fold (paranoia). Analyses indicate that the average number of mental health issues experienced after HIV diagnosis (8.71, SD 4.74) is significantly greater than that before HIV diagnosis (2.48, SD 3.89), *t*(488)=23.00, *p*<0.001.

**Table 3:** Incidence of mental health issues

	Before my HIV diagnosis, *n* (%)	After my HIV diagnosis (since/because of), *n* (%)	Extent of increase (fold)	Persistent (before and after), *n* (%)	Never, *n* (%)	Don't know, *n* (%)	Total number^a^
Depression	116 (23.9)	360 (74.0)	3.10	70 (14.4)	68 (14.0)	14 (2.9)	486
Shame	86 (17.7)	325 (70.8)	3.78	46 (10.0)	96 (20.9)	8 (1.7)	459
Self-blame	69 (14.4)	334 (70.0)	4.84	44 (9.2)	104 (21.8)	15 (3.1)	478
Very low self-esteem	107 (22.5)	292 (61.5)	2.73	57 (12)	113 (23.8)	21 (4.4)	475
Feelings of rejection	85 (18.1)	327 (69.9)	3.85	49 (10.5)	87 (18.6)	18 (3.8)	468
Body image issues	86 (18.5)	263 (56.4)	3.06	42 (9.0)	126 (27.0)	36 (7.7)	466
A strong sense of isolation	54 (11.4)	253 (53.4)	4.69	23 (4.9)	170 (35.9)	22 (4.6)	474
Paranoia	23 (5.0)	154 (33.2)	6.70	12 (2.6)	241 (51.9)	62 (13.4)	464
Anxiety	64 (13.5)	314 (66.1)	4.91	38 (8.0)	118 (24.8)	19 (4.0)	475
Insomnia	63 (13.7)	314 (68.4)	4.98	34 (7.4)	96 (20.9)	23 (5.0)	459
Anorexia	25 (5.5)	140 (30.6)	5.6	7 (1.5)	272 (59.5)	28 (6.1)	457
Difficulty going out and socializing	49 (10.4)	223 (47.4)	4.55	28 (6.0)	215 (45.7)	12 (2.6)	470
Spiritual isolation	50 (10.8)	176 (37.9)	3.52	18 (3.9)	228 (49.1)	30 (6.5)	464
Loneliness	87 (18.4)	302 (65.1)	3.47	47 (10.0)	115 (24.3)	17 (3.6)	473
Suicidal feelings	75 (15.9)	177 (37.4)	2.36	37 (7.8)	228 (48.3)	25 (5.3)	472
Post-traumatic stress disorder	69 (14.6)	177 (37.4)	2.57	36 (7.6)	182 (38.4)	33 (7.0)	474
Harmful use of drugs and/or alcohol	104 (22.0)	125 (28.5)	1.20	39 (8.3)	243 (51.5)	32 (6.8)	472

a Total *n* represents the respondents who gave an answer to each respective mental health issue question. Due to the multiple-choice design, the total number of respondents for each item does not amount to the summation of options.

As indicated in Table 2, one-third (*n*=158; 32.3%) of the respondents did not identify themselves with any SDI other than HIV. One-fifth (*n*=107; 21.9%) acknowledged one SDI (in addition to HIV). Nearly half of respondents (*n*=224; 45.8%) had multiple SDIs. Three variables, experiencing mental health issues *before*, *after* and *persistently* (both before and after HIV diagnosis), were created to understand the rate, types and changes in mental health among respondents. Table 4 shows Pearson's correlation coefficient results indicating that the more SDIs a woman living with HIV reported, the more mental health issues she would experience before diagnosis (*r*=0.19, *p*<0.01), after (*r*=0.13, *p*<0.01) and persistently (*r*=0.14, *p*<0.01).

**Table 4:** Bivariate correlations between SDIs and mental health issues with time of HIV diagnosis (*n*=489)

	SDI	Mental health issues before HIV diagnosis	Mental health issues after HIV diagnosis (since/because of)	Persistent mental health issues (before and after) diagnosis
SDI	–	0.19*	0.13*	0.14*
Mental health issues before HIV		–	0.05	0.77*
Mental health issues after HIV (since/because of)			–	0.33*
Persistent mental health issues (before and after diagnosis)				–

SDIs, socially disadvantaged identities;

* *p*<0.01.

Multiple regression analyses were conducted to examine whether socio-demographic variables (age) or SDIs were significant predictors of experiencing mental health issues before and after HIV diagnosis. Regression results are presented in Table 5. The results showed that older women with more SDIs were more likely to experience mental health issues prior to their HIV diagnosis (*F*[2,486]=11.96, *p*<0.001). In addition, women with more SDIs were more likely to report mental health issues after their HIV diagnosis (*F*=2.95 [3, 485], *p*<0.05).

**Table 5:** Multiple regression analyses of correlates of experiencing mental health issues among women living with HIV (*n*=489)

Variables	Model 1 Mental health issues before HIV diagnosis	Model 2 Mental health issues after HIV diagnosis (since/because of)
Age	0.11*	0.04
SDI	0.20***	0.13**
*R* ^2^	0.04	0.01
*F*	11.96***	2.95*

SDI, socially disadvantaged identity;

* *p*<0.05;

** *p*<0.01;

*** *p*<0.001.

### Qualitative findings

#### Stigma and mental health

While a significant proportion of respondents experienced mental health issues pre-diagnosis, women indicated that HIV diagnosis is, in itself, a flashpoint for immediate and ongoing mental health problems, especially depression, feelings of rejection and insomnia.

##### Depression

Most mental health survey respondents (82%) reported experiencing depression and depressive symptoms, often following an HIV diagnosis:

It took me well over two years to recover mentally from my diagnosis. I was withdrawn, depressed and at times suicidal. There was a lack of adequate psychological and community support services. My family and friends helped me through the worst, but I'm lucky that I have good family and friends. Many others would be totally isolated. (Ireland)

HIV-related stigma contributed to fear about disclosing to friends, which played a role in enhancing depression: “The high stigma attached to HIV infection means that I have felt very lonely – unable to fully confide in friends … This also contributed to my depression” (UK).

##### Rejection and social exclusion

Over three-quarters (78%) of survey respondents reported experiencing rejection. Respondents discussed both experiences and fears of being rejected because of HIV-related stigma. This factor was a barrier to disclosure: “I live a life of fear of disclosure and secrecy with few knowing my status and little good support” (Ireland).

This point underscores linkages between lack of disclosure and lower social support.

Respondents also discussed rejection from friends: “I have lost friends, have a hugely restricted social circle and friends from before I was diagnosed with HIV” (UK).

In other situations, family members enacted stigma:

Family member[s are] blaming me for [the] death of my husband, [I] fear [to] disclose [my status] to even my mother because she will isolate me from my siblings and use me as an example in every case. (Uganda)

Another narrative highlights coping strategies, such as exercise, employed to manage isolation:

I am again feeling very sad and [have] become a recluse again. I think HIV is a very sad and isolating disease. For all of that I will train and complete a half marathon and just will not give up. (New Zealand)

##### Sleep problems

Most survey respondents (69 to 75%) reported having experienced sleep problems and discussed many root causes, including nightmares: “During [the] night you dream things which at times if you wake up – you fear even to go out or you think maybe you can die” (Tanzania).

For some women, nightmares were associated with past trauma:

I have PTSD as a result of multiple traumas. Don't get enough sleep because I'm afraid of getting nightmares. I do fine in a crisis but after everyone is safe I shut down and don't function well. (US)

Another respondent, whose concerns for her child were an extra source of stress, also discussed this narrative of functioning all day, but having challenges sleeping at night:

I do not sleep, all sorts of thoughts go through my head, mostly related to my health. I feel sorry for myself, and for my son. He will be left alone; who will support him? And if during the day I almost never think about it, [because] I'm busy all the time, at night I cannot calm down and often cry. (Ukraine)

##### Intersectional stigma

Respondents discussed the convergence of HIV-related stigma with the stigmas surrounding other factors in their life, including those related to mental health, drug use and transgender identity. A respondent articulated how fear of disclosure due to mental health stigma can exacerbate social isolation:

Mental health problems are looked upon in a bad way by society, [they are] perceived as something that should be hidden, and most often those that struggle with mental health issues are reluctant to share with anyone resulting in further isolation. (Canada)

This was reinforced by another respondent, who described stigmas converging with sexism: “There is also stigma around mental health and it compounds HIV stigma and other forms of misogyny” (Italy).

Others discussed stigma targeting people who use drugs:

Women who use drugs are subjected to double or sometimes triple stigma. There are cases of discrimination against these women, even in [the] HIV-service organizations in which they work. (Ukraine)

Transgender stigma also emerged in service provision and laws:

Think of any service a youth could benefit from and just know that trans kids probably aren't allowed to access it. (US)

The Anti Homosexuality Act has affected trans women from getting friendly services. (Uganda)

#### Stigma, mental health issues and SRH

Respondents discussed the stigma and mental health issues that present challenges for realizing SRH. Stigma and mental health issues converged to create challenges with attaining SRH&HR in three areas: sexual and intimate relationships; subtance use and its connection with sexual risk and stigma; and, human rights (HR) violations.

##### Challenges with sexual and intimate relationships

Respondents discussed HIV-related stigma and mental health issues presenting challenges both in engaging and asserting agency and control within sexual/intimate relationships. Shame and fear were described as barriers:

I have stopped engaging in sexual relationships since being diagnosed. I feel embarrassed [and] will never disclose to anyone. (Nigeria)

The issues around dating and having to talk to your lover or partner about your status at times brings anxiety, fear and depression of being rejected. (Kenya)

As a woman explained: “It makes me less assertive and I sometimes give permission to my partner to take advantage of me by having sex when I would rather not” (Nigeria).

Another discussed a result as “little or no condom negotiation” (Jamaica).

Low self-esteem also influenced sexual relationships. A respondent articulated how low self-esteem and violence could reduce the desire to be in a relationship:

I had felt very low energy level[s] at times when my self-esteem was low, whenever I experienced violence within a marital relationship and didn't feel like getting into any relationship[s] with any men for some period in my life. (India)

By contrast, a respondent discussed how low self-esteem could result in wanting to be in a sexual relationship:

When your self-esteem is low it is very difficult to have sex because you really want to have it. You have sex because you want to be accepted, you are lonely, you just want somebody to touch you … not because you really want to. (Italy)

##### Substance use and sexual risk

Substance use was often connected to coping with mental health challenges and/or socializing. This substance use in turn was discussed in relation to sexual risk in relationships and in sex work. A respondent discussed how substance use was a way of coping with low self-esteem but led to elevated sexual risks and presented a barrier to developing relationships:

Low mood, anxiety, lack of self-esteem generally combined with enjoying alcohol with socializing (and drinking a lot when I do) resulted in putting myself at risk for HIV and hence acquisition. Ongoing low mood, anxiety etc., worsened by HIV diagnosis, encourages drinking a lot (socially) and preventing formation of close relationships. (UK)

##### Reproductive health barriers and HR violations

Respondents discussed many reproductive health barriers and HR violations, including being dissuaded from having children, mistreatment while pregnant and forced or coerced sterilization. Women who wanted to have children were often constructed as wanting to transmit the virus:

In Gabon, it is a problem to decide to have children. Women [living with HIV] who choose to do it without their doctor's advice are perceived as women who wish to transmit HIV. (Gabon)

This construction of women living with HIV as wanting to transmit the virus through childbirth resulted in some respondents unwillingly choosing not to have children:

When I found out I was HIV positive, my doctor at Planned Parenthood told me I could never have children. That I might infect them and I would be [a] “horrible woman” to do so. I didn't have children but I have regretted that decision every day of my life since. I did refuse sterilization when it was “encouraged” but still wish I had considered having children as a possibility. (US)

Some women also reported coerced and forced sterilization, which can have a profound and devastating impact on a woman's mental health:

Find a law that strongly punishes doctors performing forced sterilization and that does not expire, and [allows] time to make a lawsuit if necessary because many times we realize that we were sterilized many years later and we can do nothing. (Puerto Rico)

Others discussed childbirth mistreatment, which could prevent efforts to reduce vertical transmission:

The moment a woman identifies herself as living positively with HIV, they are neglected, especially during delivery, hence [there is an] increased number of children born with HIV because women prefer to keep it a secret and be treated like the rest. (Uganda)

#### Respondents’ recommendations

Women living with HIV recommended psychological support and counselling, peer support, challenging stigma (which is one form of GBV) and promoting HR.

##### Psychological support and counselling

Respondents recommended psychological support and counselling that was affordable, accessible, holistic and integrated. Some called for future-oriented services:

Access and encouragement to use professional mental health services. Psychologists, counsellors etc. who also have [an] understanding of the need to build a life based on well being and acceptance not over focusing on problems of the past; solution building. (UK)

Many respondents recommended integrating HIV services into a one-stop shop and that counselling should include substance use and other issues: “treat underlying causes. I was an addict & couldn't get better till I gave up all drugs. HIV was a cause/symptom of that rather than the problem” (UK).

Respondents reinforced the importance of trained, skilled provision of integrated mental health and substance use services and highlighted the need to address trauma, including “resources that actually name and explain deep rooted subconscious responses and behaviours (that are normal). Post-traumatic stress counselling” (Australia).

Underscored across many narratives was the need to increase “access to mental health services for low income women” (US), preferably without payment.

##### Peer support

Peer support was discussed as highly important in helping respondents manage mental health issues: “Connecting with other positive women was the best thing I ever did for myself” (US).

Peer support groups and networks provide: “women ‘spaces’ to be themselves, to talk about what they are going through without judgment” (UK).

This support provided hope:

Peer mentoring can be helpful. Knowing that there is life after HIV is difficult at the start of a diagnosis and anyone that can show you that there is the possibility to survive and thrive is important. (Ireland)

It was also a source of learning coping and health strategies:

Joining support groups or forums where one could meet with other infected women and learn from each other as well as how others are living with and managing their conditions/lives and medication. (Zimbabwe)

##### Challenging stigma and promoting HR

Regarding mental health experiences, women wanted policymakers “not to stigmatize women living with HIV” (Uganda) and to “end the stigma by all means possible! Policies, laws, education, public information etc. What other way is there?” (UK)

Others discussed the need to tackle “the stigma related to mental health and as well as some social norms that make mental health taboo” (Uganda).

Challenging stigma and promoting HR required policies: “real health policies addressing HIV+ women and girls issues respecting their dignity and human rights” (Brazil).

## Discussion

This largest-ever survey of women living with HIV globally examined their mental health, across the complexity of their lives and in relation to SRH&HR, GBV and treatment access combined. Women have substantial mental health issues before HIV diagnosis but experience a significant manyfold increase in the incidence of mental health issues after HIV diagnosis. Seven out of ten women report depression, shame, self-blame, feelings of rejection and insomnia, and over 50% have multiple issues. These are linked with stigma/social marginalization based on intersecting identities. SDI was a significant predictor of mental health issues before and after HIV diagnosis. This point shows the importance of examining the intersecting identities of women living with HIV and strongly corroborates the qualitative findings of intersectional stigma. Stigma (itself a form of GBV [24–27]) that targets women's identities is a key reason for the increased mental health issues post-diagnosis, highlighting the role of social and structural factors in influencing the mental health of women living with HIV.

A key strength of this original user-led and -defined study is that it examines women's perspectives of mental health before and after diagnosis with a global population. The instrument and the community-based participation were grounded in appreciative enquiry approaches [28], building on strategies and practices that work for women living with HIV. Participation was global, including women from a diverse range of ages, stages, countries and contexts, although this was not a representative sample by geography. The mixed methodology gives a comprehensive view of women's challenges in negotiating life beyond an HIV diagnosis. Due to the study design, respondents may be more connected to activist and resilient networks. They had to be mentally well enough to participate: some even reported finding it cathartic and healing. Others may have found its length and the in-depth nature of questioning too hard. Only half responded to the optional section but non-responders did not appear to have more SDIs, supporting generalizability. Although not intended as a representative sample, quantitative results thus contain selection biases and imprecision. Participation was also limited by language, Internet access and connection to networks of women living with HIV. Those in greater isolation, poverty or specific contexts may be experiencing yet greater mental health challenges, for example in areas recently affected by conflict.

In order to increase access to the survey, nine focus group discussions (FGDs) in seven countries (Ethiopia (two surveys), Jamaica, Myanmar, Nepal, Senegal (two surveys), Thailand and the United Kingdom), using five further languages (Amharic, Burmese, Nepali, Thai, Wolof), ran parallel to the online survey, using the same questionnaire (data not shown). These FGDs reached out to other women living with HIV without computers, or with limited literacy or no knowledge of the online languages used. They were facilitated by GRG members or by other women well trusted by the participants. Data analysis did not produce significant differences in responses between online survey respondents and FGD participants. This result supports the content validity of the online survey findings, since findings were not just from a relatively elite group of women with online access, but also applicable to the FGD participants.

The results are comparable with reported mental health prevalence levels of people with HIV, although only one-third of studies of interventions have included women [15]. The results are cause for concern, in terms of the complexity of these findings: the burden of HIV disease alone, conflated with the known reciprocal links between mental health, SRH&HR [8], GBV [13] and treatment access [8,14,15].

The findings indicate that clinicians and global policymakers must address the complex reciprocal effects of mental health on the lives of women living with HIV in relation to their experiences of GBV, their ability to realize their SRH&HR and their capacity to access and adhere to treatment, as a matter of urgency. Moreover, root causes of mental health issues among women living with HIV such as stigma (one pervasive form of GBV) and social marginalization need urgent attention. Given widespread HIV testing and diagnosis during pregnancy [16], itself a time of heightened potential vulnerability, and recognizing diagnosis as a critical point for mental health issues, it is especially important that future global policy guidelines regarding perinatal transmission address mental health [29].

Women living with HIV recommend that their mental health issues be addressed urgently and comprehensively in policy guidelines, in training health workers and in providing relevant services [30,31].

### Implications for research

Further research is required into the following areas: exploration into and understanding of mental health issues as a contributing factor to HIV acquisition and as a consequence of HIV diagnosis (during and outside of pregnancy and childbirth); implementation of mental health strategies in sexual health services, GBV reduction services, perinatal care and in HIV care, support and treatment; mental health issues as a significant barrier to accessing all these areas. Peer-led strategies and the effectiveness of practices that many women living with HIV find of value to manage mental health, without use of (or in combination with) relevant medication, should be tested. Links for women between conflict, mental health and HIV remain inadequately explored.

## Conclusions

Mental health challenges for women living with HIV reciprocally affect their experiences of GBV, SRH&HR and treatment access. Women living with HIV who have other SDIs can be especially affected by mental health issues. If women's mental health issues are resolved it will benefit their SRH and HR. The limited attention given in global policy guidelines to the mental health of women living with HIV as a consequence of their diagnosis is an oversight that must be addressed. Furthermore, while policymakers and clinicians clearly have a role to play, the state also needs to act more broadly and cross-sectorally to address mental health issues and social disadvantage through supportive laws and policies.

## References

[1] Murray C, Naghavi M, Wang H, Lozano R (2015). Global, regional, and national age-sex specific all-cause and cause-specific mortality for 240 causes of death, 1990–2013: a systematic analysis for the Global Burden of Disease Study 2013. Lancet.

[2] (2013). Responding to intimate partner violence and sexual violence against women. WHO clinical and policy guidelines [Internet].

[3] (1946).

[4] Kamen C, Arganbright J, Kienitz E, Weller M, Khaylis A, Shenkman T (2015). HIV-related stigma: implications for symptoms of anxiety and depression among Malawian women. Afr J AIDS Res.

[5] Prince M, Patel V, Saxena S, Maj M, Maselko J, Phillips M (2007). No health without mental health. Lancet.

[6] PUBMED US National Library of Medicine National Institutes of Health [Internet].

[7] UNAIDS (2011). Countdown to zero: global plan towards the elimination of new HIV infections among children by 2015 and keeping their mothers alive.

[8] (2006). Sexual and reproductive health of women living with HIV/AIDS: guidelines on care, treatment and support for women living with HIV and their children in resource-constrained settings [Internet].

[9] (2013). Consolidated guidelines on the use of antiretroviral drugs for treating and preventing HIV infection recommendations for a public health approach.

[10] (2015). Guideline on when to start antiretroviral therapy and on pre-exposure prophylaxis for HIV [Internet].

[11] (2010). mhGAP intervention guide for mental, neurological and substance use disorders in nonspecialized health settings [Internet].

[12] (2014). Integrating the response to mental disorders and other chronic diseases in health care systems [Internet].

[13] Orza L, Bewley S, Chung C, Crone ET, Nagadya H, Vazquez M (2015). “Violence. Enough already”: Findings from a global participatory survey among women living with HIV. http://dx.doi.org/10.7448/IAS.18.6.20285.

[14] Hatcher A, Smout E, Turan J, Christofides N, Stoeckl H (2015). Intimate partner violence and engagement in HIV care and treatment among women. AIDS.

[15] Sherr L, Clucas C, Harding R, Sibley E, Catalan J (2011). HIV and depression – a systematic review of interventions. Psychol Health Med.

[16] Kapetanovic S, Dass-Brailsford P, Nora D, Talisman N (2014). Mental health of HIV-seropositive women during pregnancy and postpartum period: a comprehensive literature review. AIDS Behav.

[17] O'Sullivan S, Thomson K (1996). Positively women.

[18] Orza L, Welbourn A, Bewley S, Crone ET, Vazquez M (2015). Building a safe house on firm ground: key findings from a global values and preferences survey regarding the sexual and reproductive health and human rights of women living with HIV [Internet].

[19] (2014). Salamander Trust values and preferences survey (Questionnaire): sexual and reproductive health and human rights for women living with HIV [Internet].

[20] (2011). In women's words: HIV priorities for positive change [Internet].

[21] (2001). Putting women first: ethical and safety recommendations for research on domestic violence against women [Internet].

[22] (2004). Guidelines on ethical participatory research with HIV positive women [Internet].

[23] Braun V, Clarke V (2006). Using thematic analysis in psychology. Qual Res Psychol.

[24] Hale F, Vazquez M (2011). Violence against women living with HIV/AIDS: a background paper [Internet].

[25] (2013). 16 ideas for addressing violence against women in the context of the HIV epidemic: a programming tool [Internet].

[26] Galtung J (1969). Violence, peace, and peace research. J Peace Res.

[27] Farmer P, Nizeye B, Stulac S, Keshavjee S (2006). Structural violence and clinical medicine. PLoS Med.

[28] McAdam E, Lang P (2009). Appreciative work in schools.

[29] Stringer EM, Meltzer-Brody S, Kasaro M, Stuebe AM, Wiegand S, Paul R (2014). Depression, pregnancy, and HIV: the case to strengthen mental health services for pregnant and post-partum women in sub-Saharan Africa. Lancet Psychiatry.

[30] Logie C, James L, Tharao W, Loutfy M (2013). Associations between HIV-related stigma, racial discrimination, gender discrimination, and depression among HIV-positive African, Caribbean, and Black Women in Ontario. Canada. AIDS Patient Care STDs.

[31] Collins PY, Holman AR, Freeman AC, Patel V (2006). What is the relevance of mental health to HIV/AIDS care and treatment programs in developing countries? A systematic review. AIDS.

